# Efficacy of goldenberry in improving obesity-induced hemoglobin conformational structure changes in wistar rats: A biophysical perspective

**DOI:** 10.1016/j.heliyon.2024.e40189

**Published:** 2024-11-07

**Authors:** Sherif A. Abdelmottaleb Moussa, Samir W. Aziz, Samir A. Bashandy, Marawan Abd elbaset, Noha A. Abd El-Latif, Sherif M. Afifi, Tuba Esatbeyoglu, Sayed A. El Toumy, Josline Y. Salib

**Affiliations:** a Biophysics Laboratory, Biochemistry Department, National Research Centre, P.O. 12622, Cairo, Egypt; b Pharmacology Department, National Research Centre, P.O. 12622, Cairo, Egypt; c Department for Life Quality Studies, Rimini Campus, University of Bologna, Corso d’Augusto 237, 47921, Rimini, Italy; d Department of Molecular Food Chemistry and Food Development, Institute of Food and One Health, Gottfried Wilhelm Leibniz University Hannover, Am Kleinen Felde 30, 30167, Hannover, Germany; e Chemistry of Tannins Department, National Research Centre, P.O. 12622, Cairo, Egypt

## Abstract

The relationship between obesity and the conformational structure of hemoglobin (Hb) has not been extensively investigated. This study aims to elucidate the dielectric parameters that distinguish the Hb molecule under obese conditions and following treatment with goldenberry (GB) extract, compared to a control group. The dielectric parameters analyzed include the loss factor (D), relaxation time (τ), dielectric increment (Δε), relative permittivity (έ), dielectric loss (ε"), conductivity (σ), and Cole-Cole parameters (α), measured across a frequency range of 20 Hz to 3 MHz. Significant differences in dielectric parameters were observed between obese rats and those treated with GB extract. Obese rats exhibited higher dielectric values compared to the control group, while rats treated with low and high doses of GB extract showed marked changes in Hb conformational structure. This study highlights the potential of dielectric parameters as biophysical markers for detecting hemoglobin conformational changes. Furthermore, it suggests that dielectric behavior could serve as an early indicator for assessing the severity of obesity and its related complications.

## 1 Introduction

Obesity is a complex, multifactorial health condition characterized by excessive fat accumulation and is associated with various comorbidities, including cardiovascular diseases, diabetes, and metabolic syndrome, all of which pose significant global health challenges [1]. Recent studies have aimed to elucidate the molecular and biochemical changes associated with obesity, with a focus on blood composition and hemoglobin (Hb) properties.

Obesity has been linked to disruptions in iron metabolism. While obesity itself typically does not lead to increased total body iron stores, it can alter the distribution of iron within the body. Adipose tissue plays a role in iron metabolism through the secretion of adipokines (cytokines produced by adipose tissue), which can modulate the expression of proteins involved in iron homeostasis. These obesity-related changes in iron metabolism may contribute to conditions such as non-alcoholic fatty liver disease (NAFLD), a disorder frequently associated with both obesity and disturbances in iron regulation [[2], [3], [4], [5]].

Hemoglobin is a crucial protein composed of two alpha and two beta subunits, each containing a heme group that binds oxygen. Its functionality relies on the ability to transition between a high-affinity relaxed (R) state and a low-affinity tense (T) state, facilitating efficient oxygen loading and unloading. In obesity, chronic hyperglycemia and oxidative stress can result in non-enzymatic glycation of hemoglobin, leading to the formation of glycated hemoglobin (HbA1c), which exhibits reduced flexibility and impaired oxygen-binding capacity [[6], [7], [8]]. Furthermore, oxidative stress may induce structural alterations in hemoglobin, such as heme iron oxidation and amino acid modifications, further compromising its function [9]. Chronic inflammation associated with obesity elevates levels of inflammatory cytokines, resulting in post-translational modifications such as nitration and phosphorylation, which disrupt hemoglobin's normal transitions between the R and T states [10]. Additionally, increased adiposity raises levels of circulating free fatty acids and lipid peroxidation products, which can interact with hemoglobin and the membranes of red blood cells, thereby affecting the conformational dynamics of hemoglobin and impairing its oxygen transport capabilities [[11], [12], [13]].

Dielectric properties offer valuable insights into the conformational states of biological macromolecules, including hemoglobin. Dielectric spectroscopy, a technique that measures the dielectric parameters of substances, can provide information about the molecular dynamics and structural changes of proteins [14]. Key dielectric parameters of interest include the loss factor (D), relaxation time (τ), dielectric increment (Δε), relative permittivity (έ), dielectric loss (ε"), conductivity (σ), and Cole-Cole parameters (α) [15]. Alterations in the dielectric properties of hemoglobin can indicate changes in its conformation and dynamics. For example, the relaxation time (τ) reflects the time scale of molecular reorientations, while the dielectric increment (Δε) is associated with the extent of polarization changes within the molecule [14]. These dielectric parameters can be influenced by factors such as protein folding, aggregation, and interactions with surrounding molecules [16].

Several studies have investigated the dielectric properties of blood and hemoglobin under various physiological and pathological conditions. Research has shown that factors such as temperature, pH, and glucose concentration can significantly affect the dielectric properties of blood [17]. In diabetic patients, changes in blood glucose levels and the presence of advanced glycation end-products (AGEs) have been linked to alterations in the dielectric properties of blood [17]. Additionally, it has been demonstrated that hemoglobin exhibits distinct dielectric behavior depending on its conformational state, such as the oxy- and deoxy-forms [14]. These findings suggest that dielectric spectroscopy could serve as a valuable tool for monitoring conformational changes in hemoglobin and their functional implications.

Goldenberry (*Physalis* *peruviana* L.) is a well-known fruit recognized for its numerous medicinal properties. Several studies have indicated that certain phytosterols specifically campesterol, β-sitosterol, and stigmasterol can lower cholesterol levels. These phytosterols are found in high concentrations in the oils extracted from *P. peruviana* L., which is known for its anti-hypercholesterolemic and antioxidant properties. Additionally, the fruit is rich in polyphenols, vitamins A and C, which contribute to its antioxidant effects [18].

In the context of obesity, this study aims to explore the dielectric parameters of hemoglobin in obese Wistar rats and investigate the potential therapeutic effects of goldenberry (GB) extract. By comparing the dielectric properties of hemoglobin across control, obese, and GB-treated groups, the study seeks to elucidate biophysical markers that may predict hemoglobin conformation and function in the context of obesity. The findings could pave the way for using dielectric behavior as an early indicator of obesity severity and its related complications.

## 2 Materials and methods

### 2.1 Plant material and reagents

In March 2021, *Physalis peruviana* fruits with husks were collected from a store in Cairo, Egypt. The plant was identified by the Herbarium of the Botany Department at the Faculty of Science, Cairo University, and voucher specimens were deposited at the National Research Center Herbarium in Cairo. The Folin-Ciocalteu reagent, prepared by mixing sodium tungstate and phosphomolybdic acid in phosphoric acid, and the AlCl_3_ reagent were obtained from Sigma (St. Louis, MO) without further modifications.

#### 2.1.1 Preparation of extract

Approximately 5 kg of fresh *Physalis peruviana* fruits with husks were washed under running water. The fruits were then lyophilized and ground using a blender. The ground material was soaked in 70 % methanol and heated to 40 °C. This extraction process was repeated three times at the same temperature. The resulting extract was filtered into a sterile flask and concentrated using a Heidolph rotary evaporator (Rothenburg ob der Tauber, Germany) at 45 °C under vacuum. The crude extract was lyophilized to obtain a dry residue weighing 60 g, which was stored in a freezer at - 4 °C until further use.

### 2.2 Animal care and treatments

Thirty-two adult female Wistar rats, weighing between 150 and 170 g, were obtained from the National Research Center Animal House in Egypt. The rats were housed in a controlled environment with a stable temperature of 25 °C and a 12-h light/dark cycle, with unrestricted access to food and water. After a one-week acclimatization period, eight rats were designated as controls, while the remaining rats were fed a high-fat diet (HFD) and provided with tap water containing 25 % sucrose for eight weeks to induce obesity. The HFD comprised 42.3 % carbohydrates, 17.0 % protein, 22.5 % fat, 3 % fiber, 2 % minerals, and 10 % moisture, while the control group was fed standard chow pellets. Animal handling procedures adhered to the guidelines set forth by the National Institutes of Health Guide for the Care and Use of Laboratory Animals and received approval from the Research Ethics Committee of the National Research Centre (approval number 19161). According to the criteria established by Novelli et al. [19], rats with a body mass index (BMI) of 0.68 or above were classified as obese. All rats in the experimental group reached the target BMI by the end of the 16-week period, and therefore, all were included in the analysis.

The rats were divided into four groups.

• Group I: Normal control rats fed a standard chow diet.
• Group II: Rats fed a high-fat diet (HFD) and tap water with 30 % sucrose for 12 weeks to induce obesity.
• Group III: Obese rats administered a low dose (200 mg/kg body weight) of Goldenberry (GB) extract orally for 8 weeks.
• Group IV: Obese rats administered a high dose (400 mg/kg body weight) of Goldenberry (GB) extract orally for 8 weeks.

### 2.3 Sample collection and hemoglobin extraction

Blood samples were collected after three months from the retro-orbital plexus under local anesthesia with phenobarbital into heparinized tubes. Plasma was separated by centrifugation at 3000*g* for 15 min. Hemolysates were prepared from washed, packed erythrocytes, and hemoglobin was extracted following a modified protocol of Trivelli et al., [16].

### 2.4 Dielectric measurements

Measurements of dielectric were performed in 20Hz to 3 MHz frequency range through a WAYNE KERR (UK) precision component analyzer model 6440B together with glass conductivity cell; model E 8071, fabricated by EDT instruments (UK). The cell sample possesses two platinum electrodes of squared black with cell constant ($k=1\;cm^{-1}$). Dielectric measurements of hemoglobin (Hb) of the control, obese groups, and after treatment with low and high dose of GB groups were done at 20 °C.

The capacitance C and resistance R values were measured when the dielectric material is placed between two parallel plates capacitor, to calculate the conductivity σ, the real εʹ, the imaginary ε", the complex permittivity $\varepsilon^{*}=\varepsilon^{\prime }-j\varepsilon^{\prime \prime }$, and the relaxation time τ through the following equations:

i) $\varepsilon^{\prime }=\frac{C}{C_{o}}=\frac{C}{\varepsilon_{o}}k=\frac{C}{\varepsilon_{o}}\left(\frac{d}{A}\right)=\frac{C}{8.85\times 10^{-12}}\times 100=1.13\times 10^{13}C$

Where (d/A) is the cell constant (k = 1 cm^−1^) that depends on the cell dimension.

ε_o_ is the permittivity of the free space.

ii) The loss tangent factor $D=\frac{\varepsilon^{\prime \prime }}{\varepsilon^{\prime }}=\frac{1}{2\pi fRC}$
iii) The dielectric loss $\varepsilon^{\prime \prime }=D\varepsilon^{\prime }$
iv) The conductivity $\sigma =\frac{1}{\left(R\frac{A}{d}\right)}=\frac{k}{R}=\frac{100}{R}\left(\Omega^{-1}m^{-1}\right)$
v) The dielectric increment $\Delta \varepsilon =\varepsilon_{1}-\varepsilon_{o}$
vi) The relaxation time $\tau =\frac{1}{2\pi f_{c}}$, f_c_ is the critical frequency corresponding to the mid-point of the dispersion curve.
vii) The plot ε" vs. ε' (Cole-Cole plot) will result a semi-circle. Cole and Cole show that the angle drawn between the radius of the circle and the axis ε′ is $\theta =\frac{\pi \alpha }{2}$. This in turn allows estimation of the Cole-Cole parameter α, experimentally, $\alpha =\frac{2\theta }{\pi }$.

### 2.5 Statistical analysis

Each value is expressed as mean and standard error (SE). One-way analysis of variance (ANOVA) was utilized to compare each variable in the different calculated groups. For all statistical comparisons a value of p < 0.01 was considered significant.

## 3 Results

The dielectric increment, relaxation time, molecular radius, and Cole-Cole parameter (α) of hemoglobin (Hb) molecules were calculated for all groups and are presented in Table 1. The molecular radius was derived from the relaxation time data. As indicated in Table 1, the dielectric increment, relaxation time, Cole-Cole parameter, and molecular radius of Hb increased in the obese rats compared to the control group. However, a significant decrease was observed in the groups exposed to both low and high doses of GB extract. The variations in the dielectric parameters among the studied groups can be attributed to changes in the relaxation time of the Hb molecules. Based on the current data, it can be concluded that the dielectric characteristics of hemoglobin fall within α dispersion region. These results demonstrate a broad distribution of relaxation times for the Hb molecules in the obese group relative to the control group. Notably, after GB supplementation, whether at low or high doses, the molecular relaxation times of Hb became similar to those of the control group.

**Table 1:** Dielectric dispersion (Δε), relaxation time (τ), molecular radius, angle (θ, subtended between the radius of curvature and the ε′ axis), and the Cole-Cole parameter (α = 2θ/π) of hemoglobin for the Obese group, Obese + Low Dose of GB Extract group, and Obese + High Dose of GB Extract group compared to the Control group.

Groups	Parameter				
	Δε x10^−3^	τ $=\frac{1}{2\pi f_{c}}$ (10^−3^s)	Molecular radius (nm)	ϴᵒ	α x 10^−4^
**Control**	0.453 ± 5.7	0.260 ± 5.1	3.41 ± 0.02	1.009	0.012 ± 3.8
**Obese**	0.671 ± 3.5	0.371 ± 3.5	4.12 ± 4	8.432	0.101 ± 5.6
**Obese + Low dose of GB**	0.513 ± 2.7	0.311 ± 2.4	3.78 ± 3.7	5.146	0.005 ± 2.6
**Obese + High dose of GB**	0.488 ± 2.3	0.296 ± 2.1	3.52 ± 2.6	2.155	0.011 ± 2.1

**Fig. 1:**
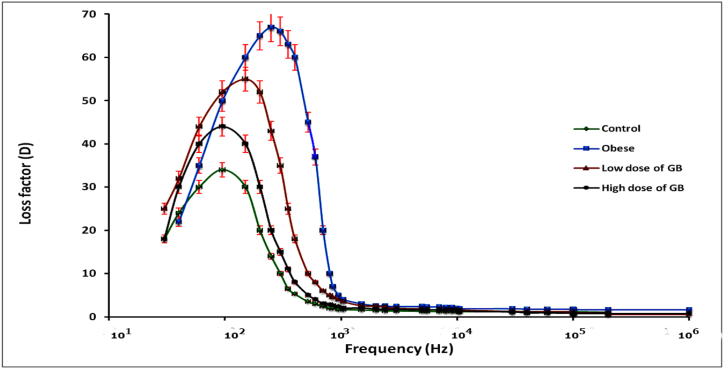
Variation of the loss factor (D) as a function of frequency (Hz) in the 20 Hz to 3 MHz range for the Obese group, Obese + Low Dose of GB Extract group, and Obese + High Dose of GB Extract group, compared to the Control group.

A higher loss factor (D) was observed in the obese group, with a subsequent decrease in the loss factor for both the low and high dose GB groups compared to the control group (Fig. 1). These results indicate that hemoglobin (Hb) molecules exhibit dielectric dispersion within the specified frequency range, as evidenced by the observed dielectric increment ($\Delta \varepsilon =\varepsilon_{1}-\varepsilon_{0}$).

**Fig. 2:**
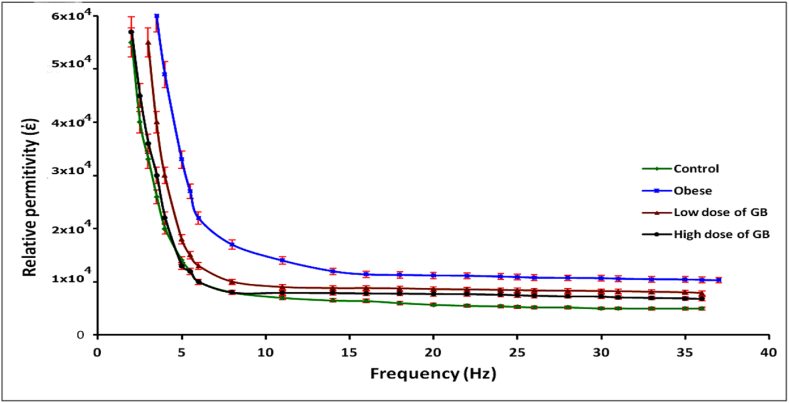
Variation of relative permittivity as a function of frequency (Hz) in the range of 20 Hz to 3 MHz for the Obese, Obese + low dose of GB extract, and Obese + high dose of GB extract groups, compared to the control group.

Fig. 2 showed a significant increase in the relative permittivity values for the obesity group and a substantial decrease in these values for the groups subjected to low and high doses of GB sequentially, compared to the control group.

**Fig. 3:**
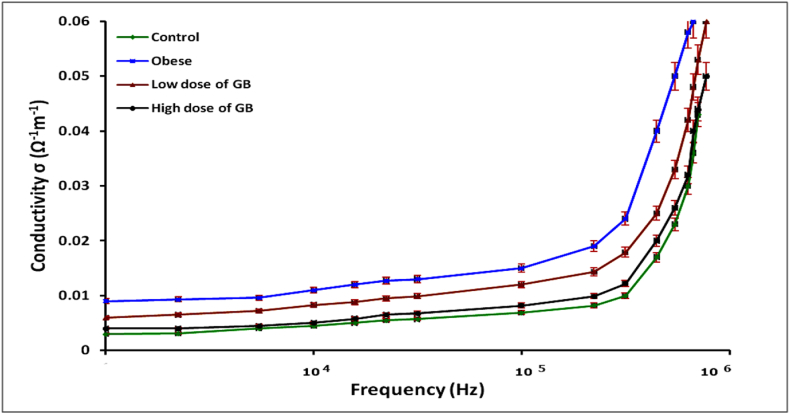
Variation of conductivity as a function of frequency (Hz) in the 20 Hz to 3 MHz range for the Obese group, Obese + Low Dose of GB Extract group, and Obese + High Dose of GB Extract group, compared to the Control group.

The results shown in Fig. 3 indicate that the groups exposed to both low and high doses of GB extract exhibited lower conductivity values compared to the control group. In contrast, the obese group displayed higher conductivity values.

**Fig. 4:**
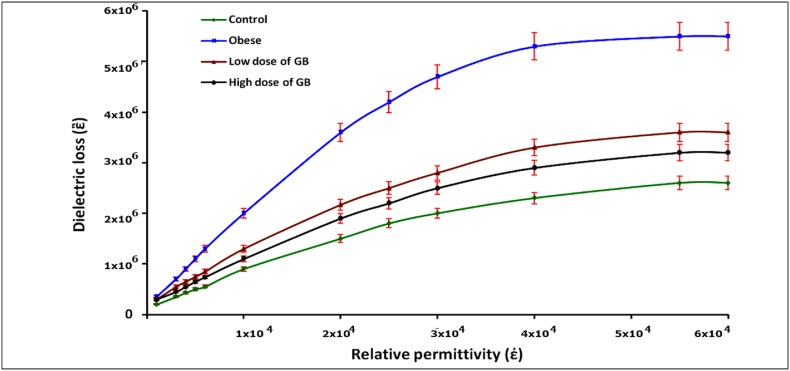
Variation of dielectric loss as a function of relative permittivity for the obese group, Obese + Low Dose of GB Extract group, and Obese + High Dose of GB Extract group, compared to the Control group.

The variance in dielectric loss as a function of the relative hemoglobin permittivity was higher for the obese group compared to the control group, while decreasing values were evident for both the low and high dose GB groups (Fig. 4).

**Fig. 5:**
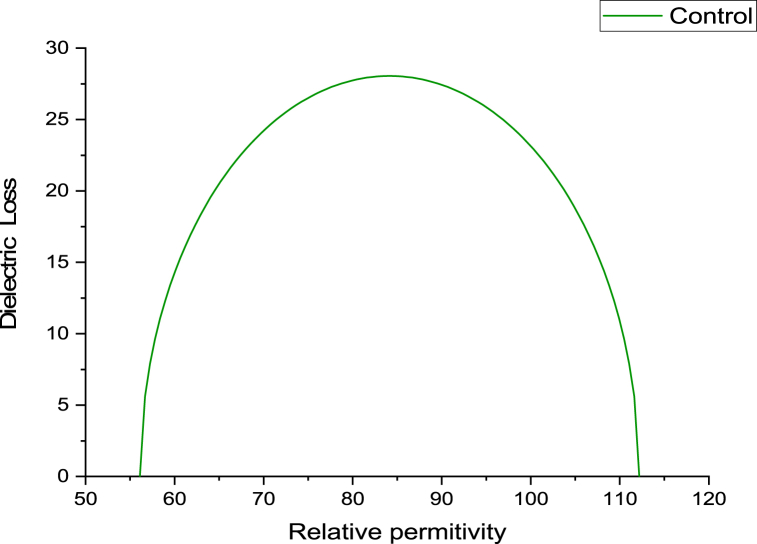
Cole-Cole plot for hemoglobin of control group.

**Fig. 6:**
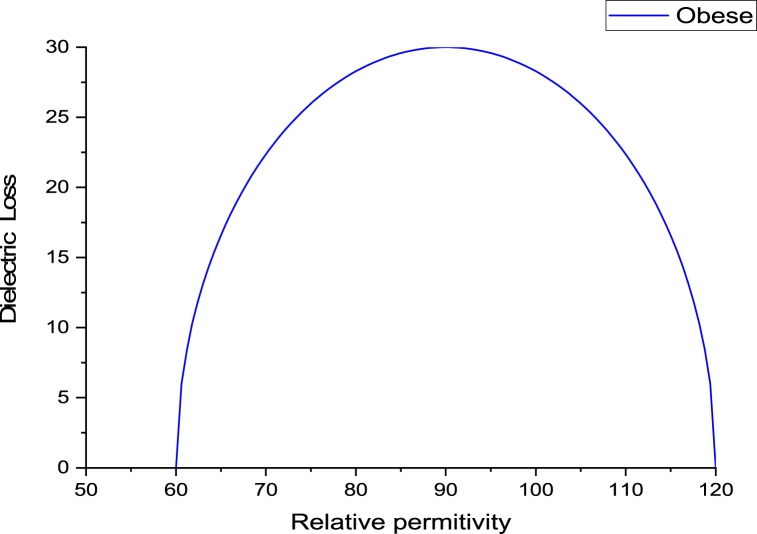
Cole-Cole plot for hemoglobin of obese group.

**Fig. 7:**
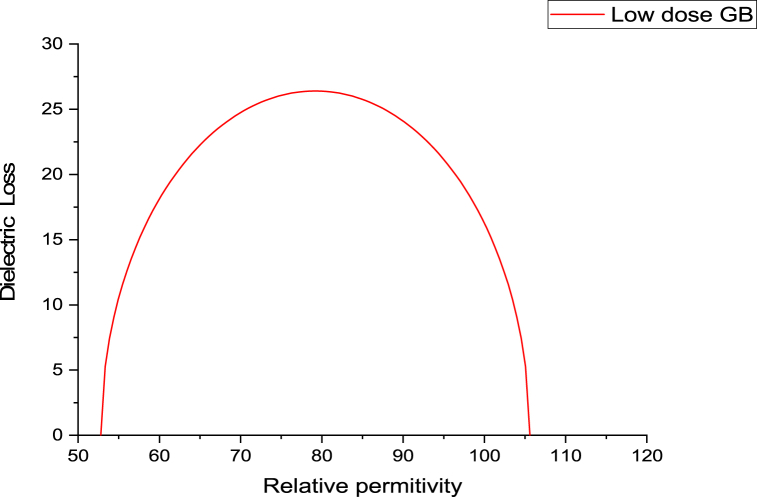
Cole-Cole plot for hemoglobin obese + low dose GB group.

**Fig. 8:**
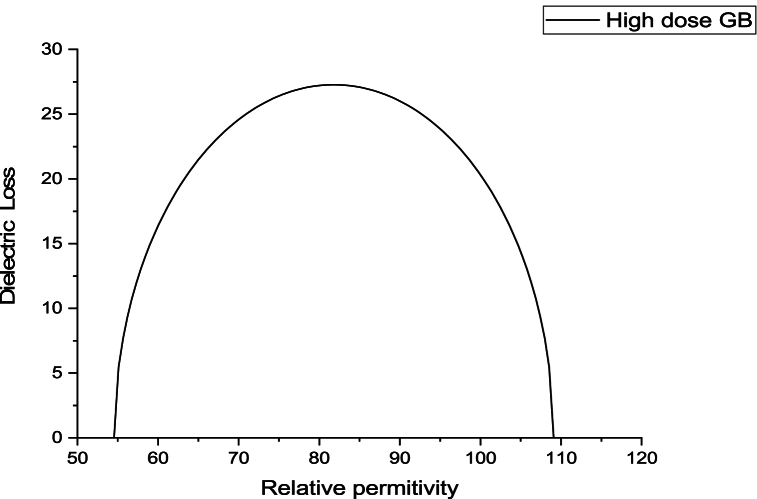
Cole-Cole plot hemoglobin of obese + high dose GB group.

Fig. 5, Fig. 6, Fig. 7, Fig. 8 present Cole-Cole plots for four groups: the control group, the obese group, the obese group with a low dose of GB extract, and the obese group with a high dose of GB extract. From these figures, the values of the Cole-Cole parameter (α) for all samples were deduced and summarized in Table 1. The results indicate a significant distribution of relaxation times of hemoglobin molecules in the obese group compared to the control group. In contrast, both the low and high dose GB extract groups exhibited reduced values of α. Curve fitting analysis demonstrated that the Cole-Cole model provided a superior fit for the dielectric data.

## 4 Discussion

Obesity induces systemic oxidative stress through various biochemical mechanisms, including superoxide generation by NADPH oxidases, oxidative phosphorylation, glyceraldehyde auto-oxidation, and activation of protein kinase C. Additional contributors to oxidative stress in obesity include hyperleptinemia, reduced antioxidant defenses, chronic inflammation, and the generation of reactive oxygen species following meals [10].

The metabolic, inflammatory, and oxidative stress changes associated with obesity can significantly affect the molecular structure and dynamics of proteins, such as hemoglobin. Increased oxidative stress may lead to alterations in protein structure, potentially influencing properties like size (radius) and interaction with their environment (relaxation time) [20].

In this study, we examined the conformational changes of hemoglobin using dielectric relaxation across a frequency range of 20 Hz to 3 MHz, which encompasses the α and β dispersion frequencies. This phenomenon, known as frequency dispersion, is observed in various biological materials [21]. Dielectric relaxation provides insights into biophysical properties such as relaxation time, shape, and molecular radius of the hemoglobin molecule.

The data presented in Table 1 indicate that both the relaxation time and radius of hemoglobin (Hb) molecules significantly increased in the obese group, by 42.69 % and 20.95 %, respectively. In contrast, the low-dose GB group showed increases of 19.61 % and 10.81 %, while the high-dose GB group exhibited increases of 13.84 % and 3.22 %. The elevated relaxation time and radius in the obese group likely reflect changes in molecular size, indicative of an unfolding process observed during β dispersion (see Fig. 1 and Table 1). Generally, shorter relaxation times and larger critical frequencies are associated with smaller molecules [22].

Obesity, which is characterized by chronic low-grade inflammation and oxidative stress, may induce modifications in protein structure and function, including that of hemoglobin. Increased oxidative stress can lead to alterations in hemoglobin molecules, impacting their physical properties such as relaxation time and size. Given that hemoglobin plays a critical role in oxygen transport, any changes in its structure could affect its functionality [20,23].

The dielectric increment increased significantly in the obese group (48.12 %) compared to the low-dose GB group (13.24 %) and the high-dose GB group (7.72 %) relative to the control group. This suggests that obesity alters the dielectric properties of the body, potentially due to changes in fat distribution, hydration levels, or metabolic factors associated with obesity. Our findings are consistent with previous studies by Aziz et al. [24], which indicate that obesity impacts hemoglobin conformation.

The Cole-Cole parameter (α) of hemoglobin (Hb), calculated for the obesity and GB groups relative to controls, indicates conformational changes in hemoglobin. The α values show a wide distribution, ranging from 0.012 in the control group to 0.101 in the obesity group. Obesity may cause the shape of the Hb molecule to deviate from spherical due to an unfolding process, thereby affecting its hydrophobic/hydrophilic ratio [25,26].

Additionally, the relative permittivity of Hb increased in the obesity group compared to the controls (Fig. 2). This increase is likely attributed to a higher surface charge density resulting from hydroxyl, superoxide, hydrogen peroxide radicals, and lipid peroxides generated during obesity. The oxidative stress associated with obesity chemically alters Hb, leading to functional and structural modifications [27]. Furthermore, obesity affects body composition and fluid dynamics, potentially altering the dielectric properties of tissues and blood where hemoglobin is present [28].

The Cole-Cole plot of hemoglobin reflects its dielectric properties, which are influenced by the molecular radius. Larger hemoglobin molecules exhibit distinct relaxation behaviors that can be identified in the shape and dispersion of the Cole-Cole plot. This analysis provides valuable insights into their size and molecular interactions [29].

The Cole-Cole plot of hemoglobin is nearly semicircular. However, in obesity, the shape of the hemoglobin molecule tends to deviate from its spherical form. Obesity leads to significant structural and functional changes in hemoglobin, including increased glycation, oxidative modifications, altered oxygen affinity, and elevated levels of hemoglobin. These changes contribute to impaired oxygen transport and may exacerbate cardiovascular and metabolic complications [[30], [31], [32], [33]].

As shown in Fig. 5, Fig. 6, Fig. 7, Fig. 8, there is an increase in the unfolding of hemoglobin, with varying degrees of unfolding as a globular protein correlating with changes in the hydrophobic/hydrophilic ratio. This alteration in the tertiary structure of the hemoglobin molecule results in a shift in its molecular shape from nearly spherical to a non-spherical form, reflected in different values of the parameter α.

In summary, goldenberries demonstrate potential biophysical effects on hemoglobin in the context of obesity, particularly in addressing iron overload and oxidative stress. Obesity induces oxidative stress and inflammation, leading to structural and functional changes in proteins like hemoglobin. Antioxidant-rich substances, including phenolics and flavonoids, contribute to reducing blood lipid peroxidation and increasing levels of antioxidants such as superoxide dismutase (SOD) and glutathione (GSH). These factors help protect red blood cells from damage, improving the overall health of hemoglobin and highlighting their potential as anti-obesity agents [34,35].

## 5 Conclusion

This research ultimately aims to establish dielectric parameters that can detect disease-induced changes in the electrical properties of the hemoglobin (Hb) macromolecule. Additionally, dielectric measurements of Hb macromolecules may serve as predictive indicators of obesity and its associated complications. However, the morphological changes of hemoglobin under this treatment should be further characterized using modern experimental instruments in the future to support the significance of electrical parameter measurement.

## CRediT authorship contribution statement

**Sherif A. Abdelmottaleb Moussa:** Writing – review & editing, Writing – original draft, Conceptualization. **Samir W. Aziz:** Writing – review & editing, Writing – original draft. **Samir A. Bashandy:** Data curation. **Marawan Abd elbaset:** Data curation. **Noha A. Abd El-Latif:** Methodology. **Sherif M. Afifi:** Resources, Project administration, Funding acquisition. **Tuba Esatbeyoglu:** Writing – review & editing, Funding acquisition. **Sayed A. El Toumy:** Methodology. **Josline Y. Salib:** Methodology.

## Ethics approval and consent to participate

The National Research Centre, Medical Research ethics committee has approved all procedures and experiments with permit no. “19161”.

## Data availability

Data will be available upon request from Sherif A. Abdelmottaleb Moussa.

## Funding

This research receives financial support from National research Centre under grant number [12060172]. The publication of this article was funded by the Open Access Fund of Leibniz Universität Hannover.

## Declaration of competing interest

The authors declare that they have no known competing financial interests or personal relationships that could have appeared to influence the work reported in this paper.
